# Editorial: Mobility in older adults with cognitive impairment

**DOI:** 10.3389/fnagi.2023.1257605

**Published:** 2023-07-25

**Authors:** Bård Bogen, Gro Gujord Tangen, Riona Mc Ardle

**Affiliations:** ^1^Department of Health and Functioning, Western Norway University of Applied Sciences, Bergen, Norway; ^2^Department of Rehabilitation Services, Haraldsplass Deaconal Hospital (HDS), Bergen, Norway; ^3^Norwegian National Advisory Unit on Ageing and Health, Vestfold Hospital Trust, Tønsberg, Norway; ^4^Faculty of Medical Sciences, Newcastle University, Newcastle upon Tyne, United Kingdom

**Keywords:** gait, mobility, older, cognitive, dementia

“If you want to know if your brain is flabby, feel your legs.”

The quote is attributed to politician, advertising executive and author Bruce Barton (https://en.wikipedia.org/wiki/Bruce_Fairchild_Barton) and frames the topic at hand: the link between motor and cognitive performance. Over the last number of decades, there has been a growing focus on this in both research and clinical practice, with the aim of finding early biomarkers or diagnostic signs, or identifying therapeutic pathways for cognitive impairment. Given the expected increase of older people living with dementia, this is timely (Prince et al., 2015). In this Research Topic, we called for papers exploring the link between mobility and cognitive impairment in older adults. Being mobile, i.e., being able to walk or transport oneself safely and efficiently, is vital for independence and wellbeing, as well as for connecting our physical, mental, social and emotional needs with our sense of self (Rantakokko et al., 2013; Delgado-Ortiz et al., 2023). Additionally, gait and mobility are useful actions to analyze, as multiple (if not all) organ systems and body segments are involved (Hausdorff, 2005).

Several world class researchers have contributed to this Research Topic, including research on cognitively healthy older participants, patients with stroke and patients with neurodegenerative disorders such as Alzheimer's and Parkinson's disease. Some have explored biomarkers and neuroimaging markers and their associations with mobility, others have investigated changes in cognition and mobility over time. One paper addresses the possible mediators of the link between cognition and mobility, and one paper explores a possible new gait measure and the association with cognition.

Tangen et al. (*Mobility and associations with levels of cerebrospinal fluid amyloid* β *and tau in a memory clinic cohort*) investigated cerebrospinal fluid [amyloid -β 42 (Aβ_42_), total tau (t-tau) and phospho tau (p-tau_181_)] in older adults in a memory clinic, with subjective and mild cognitive impairment, and dementia. Mobility was assessed with gait speed, Mini-BESTest, Timed “Up & Go” (TUG) and TUG dual task-cost (TUG DTC). There were significant associations between Aβ42 and all mobility outcomes, and the strongest association was seen with the Mini-BESTest. This suggests that dynamic balance can be closely related to Alzheimer's specific pathology.

Einstad et al. (*Neuroimaging markers of dual impairment in cognition and physical performance following stroke: the Nor-COAST study*) looked at data from the Nor-COAST cohort. Both cognitive and physical performance is known to possibly deteriorate after stroke. The aim of the study was to investigate whether pre-stroke pathology was associated with dual decline (both physical and cognitive decline). Secondly, they wanted to investigate if white matter hyperintensities (WMH), medial temporal lobe atrophy and stroke lesion location and volume were associated with dual decline. The authors found that dual decline was rather common, and that stroke lesion volume was associated with dual decline.

Geritz et al. (*Cognitive parameters can predict change of walking performance in advanced Parkinson's disease—Chances and limits of early rehabilitation*) investigated how cognitive and affective parameters could predict gait changes in patients with Parkinson's disease after 2 weeks of rehabilitation. Global cognition (Montreal Cognitive Assessment; MoCA) executive functions and divided attention (Trail making test B minus A; ΔTMT), depressive symptoms and fear of falling was assessed at baseline. Gait parameters were estimated using body-worn inertial sensors during single (ST) and dual task walking. During ST, low performance at ΔTMT was associated with lower reduction in step time asymmetry. During dual task walking, low performance at ΔTMT was associated with reduced stride time and lower double limb support. Higher MoCA at baseline was associated with higher gait speed after rehabilitation.

Beauchet et al. (*New onset, transient and stable motoric cognitive risk syndrome: clinical characteristics and association with incidence of probable dementia in the NuAge cohort*) investigated new onset, stability and transience of motoric cognitive risk syndrome (MCR) over 1 year, and association with incident dementia, in the NuAge cohort of community-living older adults. They found a prevalence of MCR of 8.5%, with 4.3% having a new onset, 2.8% were transient and 1.4% were stable. A higher score on the Geriatric Depression Scale (GDS) was associated with risk of new onset and transient MCR, and cerebrovascular disease was more frequent in stable MCR than in non-MCR. MCR was overall associated with higher incidence of probable dementia.

Chen et al. (*Mediating effect of lower extremity muscle strength on the relationship between mobility and cognitive function in Chinese older adults: a cross-sectional study*) investigated cognitive function using the Mini Mental State Examination (MMSE), mobility using the Timed “Up & Go” (TUG) and knee extensor strength in a cohort of older adults. The aim was to estimate the role of muscle strength in the relationship between cognitive function and mobility. Mobility and cognitive function were significantly correlated. They further found significant correlations between mobility (TUG) and muscle strength, and between cognition and muscle strength. The authors estimated that muscle strength had a mediating effect of approximately 20% in the correlation between mobility and cognitive function.

Knapstad et al. (*The association between cognitive impairment, gait speed, and Walk ratio*) looked at the association between gait parameters and cognitive function. The Walk ratio (step length divided by step frequency) is assumed to be a measure of central gait control. In a literature review, the authors found that the Walk ratio was significantly associated with cognitive function measured with the Mini-Mental State Examination (MMSE). However, gait speed had a closer association with cognition, and may be a preferable parameter to the Walk ratio in assessment of mobility of cognitively impaired older adults.

In summary, in this Research Topic, steps have been taken to further untangle the link between how we move and think. Further research efforts are needed to establish diagnostic and therapeutic pathways building on the rapidly accumulating evidence on this topic.

## Author contributions

All authors listed have made a substantial, direct, and intellectual contribution to the work and approved it for publication.

## References

[B1] Delgado-Ortiz L. Polhemus A. Keogh A. Sutton N. Remmele W. Hansen C.. (2023). Listening to the patients' voice: a conceptual framework of the walking experience. Age Ageing 52, 1. 10.1093/ageing/afac233 36729471 PMC9894103

[B2] Hausdorff J. M. (2005). Gait variability: methods, modeling and meaning. J. Neuroeng. Rehabil. 2, 19. 10.1186/1743-0003-2-19 16033650 PMC1185560

[B3] Prince M. W. A. Guerchet M. Ali G.-C. Wu Y.-T. Prina M. (2015). “World Alzheimer Report 2015,” in The Global Impact of Dementia. An Analysis of Prevalence, Incidence, Cost and Trends. London: Alzheimer's Disease International.

[B4] Rantakokko M. Mänty M. Rantanen T. (2013). Mobility decline in old age. Exerc. Sport Sci. Rev. 41, 19–25. 10.1097/JES.0b013e3182556f1e 23038241

